# Silver nanoparticle-induced alteration of mitochondrial and ER homeostasis affects human breast cancer cell fate

**DOI:** 10.1016/j.toxrep.2022.10.017

**Published:** 2022-11-01

**Authors:** Smita Dey, Leena Fageria, Ankita Sharma, Sudeshna Mukherjee, Surojit Pande, Rajdeep Chowdhury, Shibasish Chowdhury

**Affiliations:** a Department of Biological Sciences, Birla Institute of Technology and Science (BITS), Pilani, Pilani Campus, Rajasthan 333031, India; b Department of Chemistry, Birla Institute of Technology and Science (BITS), Pilani, Pilani Campus, Rajasthan 333031, India

**Keywords:** Silver nanoparticles, Mitochondria, Endoplasmic reticulum

## Abstract

Breast cancer is one of the most frequent forms of cancer. Although different treatment modalities are available, none has proved to be a game-changer. In this context, nanomedicine is one of the hot research areas, with different nano-formulations being explored as a therapeutic strategy against breast cancer. Herein, silver nanoparticles (AgNPs) have shown prospects with their anti-tumor properties and are currently being explored aggressively; however, the underlying molecular mechanisms of AgNP action remain to be unearthed. As part of this study, human breast cancer cells- MCF7 were exposed to AgNPs (∼9 nm), and the effect of the same was explored on mitochondrial and endoplasmic reticulum (ER) dynamicity. We observed that the AgNPs co-localize with mitochondria and cause mitochondrial membrane depolarization, ROS generation, and destabilized mitochondrial homeostasis. Also, the NPs were found to enhance ER stress. We further found that increased ER stress is linked to the disruption of mitochondrial dynamics. Overall, our study shows that the AgNPs can effectively cause apoptosis of MCF-7 cells by regulating the mitochondrial-ER dynamicity. The results provide an insight into the mechanisms via which AgNPs act and can be used in developing a potential chemotherapeutic agent.

## Highlights

• AgNPs enter mitochondria, cause membrane depolarization and mitochondrial ROS generation.
• AgNPs cause mitochondrial fragmentation and fission.
• AgNPs induces ER stress and disrupt ER morphology.
• Increased ER stress leads to more mitochondrial damage and cell death.

## Graphical Abstract

**Figure:**
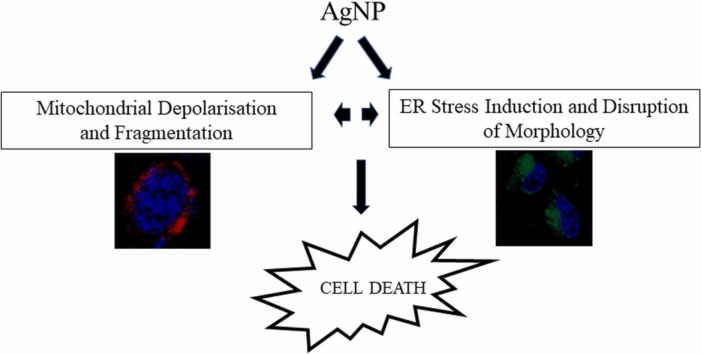

## 1 Introduction

Breast cancer is a prevalent form of cancer across the globe [1]. Different treatment modalities are in vogue based on the diagnosis, stage, and type of breast cancer. However, the fatality ratio is still a major cause of concern, indicating the requirement for a novel effective therapy [2]. In this context, we believe that cancer nanomedicine can revolutionize the way cancer is currently treated. In the past decade, research using nanoparticles (NPs) for treating different cancers has shown considerable progress. NPs provide several advantages, including improved targetability, cell-specific toxicity, enhanced permeability and retention (EPR), effect and augmented intracellular localization [3], [4]. Moreover, several NPs have also been reported to target specific cell organelles, thus increasing their efficacy as an anti-tumor modality.

In this context, mitochondria have gained much attention because of their involvement in shaping cellular metabolism and energy requirement. Mitochondria can undergo dynamic changes, and this property of mitochondria is often capitalized by tumor cells for their benefit. For example, mitochondria undergo repetitive cycles of fusion or fission, which have evolved as a protective mechanism in response to diverse stress stimuli, including tumor cell survival under chemotherapeutic shock. However, it is much debated how tumor cells combat the stress stimuli or meet their high energy demand through precise regulation of mitochondrial dynamics. There are many theories to it- for example, according to Warburg’s hypothesis, the cancer cells depend mainly on glycolysis and not on mitochondrial respiration [5]; At the same time, another group of scientists reports that tumor cells have well-functional mitochondria and have even shown an increased amount of mitochondrial biogenesis, which includes the fusion of the existing mitochondria. Precisely, the tumor cells can efficiently regulate the cellular quality control system continuously through stringent regulation of mitochondrial dynamicity and meet their proliferative needs [6]. Considering the role of mitochondria, various studies have focused on targeting mitochondria in treating cancer. The mitochondria targeting drugs like Venetoclax and Azacitidine (oxidative phosphorylation suppressor) [7], Rapamycin (mTOR inhibitor), SB-204990 (ATP citrate lyase chemical) [8], Metformin, IACS-010759, Rotenone, Oligomycin, Gboxin (complex I inhibitors) [9] are used in different cancer treatment. However, further studies are required in this direction to develop a fool proof approach against tumor types that can show intra-tumor genetic and physiological heterogeneity.

Another organelle with intense crosstalk with mitochondria and playing a vital role in maintaining cellular homeostasis is the endoplasmic reticulum (ER). It is the primary site for protein folding and calcium storage [10]. Interestingly, ER has emerged as an organelle that is reported to contribute to the regulation of mitochondrial integrity and functionality as well [11]. Mitochondria and ER membranes are interlinked with each other, maintaining equilibrium, both functionally and structurally, through tight junctions regulated by a channel of molecular connectivity formed at certain subdomains, which are also called mitochondria-associated membranes (MAM) [12]. Furthermore, disturbed proteostasis within a cell can give rise to ER stress, a cascade of events including unfolded protein response (UPR) and ER-associated degradation (ERAD); this stress can, in turn, also alter the organelle dynamicity, especially affecting the integrity of mitochondria [13]. The stimulation of UPR can also play a protective function in tumor cells. Thus existing cumulative evidence show interdependence between mitochondria and ER [10], giving multiple reasons why a thorough study is important to explore the possible crosstalk between these organelles under specific cytotoxic stress.

In the current research, we report how AgNPs affect organelles like mitochondria, ER, and their crosstalk in breast cancer cells. The changes in organelle dynamicity, especially mitochondria and ER, have been monitored and correlated with the cytotoxicity of AgNPs.

## 2 Materials and methods

### 2.1 Chemicals and reagents

Tunicamycin (Tuni, #sc-3506) and JC-1 (#sc-364116A) were purchased from Santa Cruz Biotechnology. RIPA Buffer (#R0278), Protease Inhibitor Cocktail (PIC, #P8340), Propidium Iodide (PI, #P4864) were purchased from Sigma; 3- [4, 5-dimethylthiazol-2-yl]− 2,5-di-phenyltetrazolium bromide (MTT, #33611) was obtained from SRL; beta-cyclodextrin (β-CD, #C0900), and 3-(2-Benzothiazolyl)− 7-(diethylamino), coumarin (C6, #B2088) were purchased from TCI Chemicals. MitoSOX (#M36008), Fura-2 AM cell permeant(#F1221), ER Tracker Green (#E34251) was purchased from Invitrogen. FITC conjugated AnnexinV (#A13199), AnnexinV binding buffer (#V13246), Enhanced Chemiluminescence (ECL, #32106) and Antifademountant (4′-6-diamidino-2-phenylindole, #P36962) were procured from Thermo Fisher Scientific. Antibodies (TOM20 #D8T4N, DRP1 #D6C7, phospho-DRP1 #D9A1, IRE1α #14C10, BiP #C50B12, Calnexin #C5C9) were obtained from Cell Signaling Technology (CST, USA) and Santa Cruz Biotechnology (β-actin #sc-69879, GAPDH #sc-365062) and secondary antibodies- (anti-mouse #7076 S and anti-rabbit #7074P2) were procured from Cell Signaling Technology.

### 2.2 Synthesis of AgNP

**Fig. 1:**
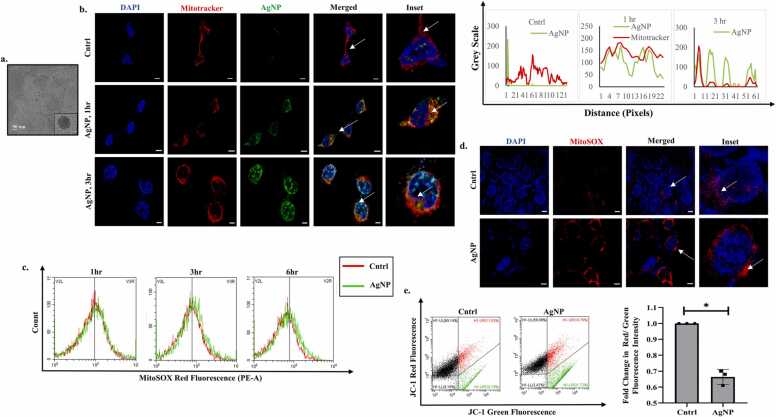
AgNP localizes in the mitochondria & affects mitochondrial health (a)TEM image of 9 nm AgNP. Inset figure represents HRTEM image of AgNP. Scale bar represents 50 nm. (b) Fluorescence images captured after exposure to Coumarin-6 tagged AgNPs (25 μM). Mitochondria are stained using Mitotracker Red. The scale bar represents 10 µm. The adjacent graph represents the fluorescent intensity profile for co-localization as analyzed from the microscopic image. (c) Analysis of mitochondrial ROS using MitoSOX Red upon AgNP (25 μM) treatment as analysed through flow cytometry. (d) Fluorescence images representing mitochondrial ROS post-AgNP (25 μM) exposure for 6 hr. Scale bar represents 10 µm. (e) Analysis of mitochondrial membrane potential by JC-1 post-AgNP (25 μM) treatment for 6 hr. Data are expressed as mean ± SD. * indicates the significant difference from control calculated using the student’s t-test (p < 0.05).

AgNP was developed following our previously reported method [14]. In brief, 0.16 g β-cyclodextrin (β-CD) was dissolved in Milli-Q water (5 mL) and 300 μl NaOH (1.0 M) was added to make the solution alkaline (pH ∼ 10). After that, the metal salt, AgNO_3_ (10 μl of 0.1 M) was added and mixed properly. Finally, the reaction mixture was kept in a water bath at 85 ⁰C. A yellow color solution appeared after ∼ 10 min, which confirms the generation of AgNP. To check the size of AgNP, transmission electron microscope (TEM) analysis was performed, and the average size was reported as 9 nm (Fig. 1a).

### 2.3 Cell culture

Breast cancer cells-MCF-7 cell was procured from NCCS (Pune, India). Cells were cultured in Dulbecco’s modified eagle medium (HIMEDIA) supplemented with 10% Fetal bovine serum (HIMEDIA) and antibiotics (1% penicillin-streptomycin) at 37º C in 5% CO_2_.

### 2.4 Immunoblotting

Small size silver nanoparticles (AgNPs) (approximately 9 nm) were chemically formulated, and the MCF7 cells were treated with these AgNPs or drug plus AgNPs for 6 hr, and Immunoblotting was performed according to the method described earlier [14]. The blots were probed with specific antibodies at a dilution of 1:1000, and the housekeeping gene, GAPDH, and β-actin in the dilution of 1:2000 was used as a loading control. The horseradish peroxide conjugated secondary antibody was used in the dilution of 1:5000, and the intensity of the protein was determined using Enhanced Chemiluminescence by Chemi-Doc XRS (Bio-Rad) detection system, and the protein expression level was determined densitometrically, using the tools of ImageJ 1.53a and Image Lab 6.1 software.

### 2.5 Immunofluorescence

The MCF7 cells were seeded on the coverslip and were treated with AgNPs for 0 hr, 1 hr, and 6 hr, respectively. The cells were fixed following the process explained in *Chowdhury* et al. [15]. For blocking, 2.5% BSA was used and incubated for 2 hr. Overnight incubation of anti-rabbit monoclonal primary antibody at 4º C, followed by 2 hr incubation of FITC conjugated and Alexa-Fluor 488 conjugated secondary antibody from Santacruz and Invitrogen, respectively, was performed in the following steps. The coverslips were mounted with the antifading agent DAPI, and finally, the expression of the proteins was visualized through ZEISS Axio Scope A1 Microscope.

### 2.6 Co-localization of AgNPs with mitochondria

To determine the co-localization of the AgNPs inside the mitochondria, the AgNPs were first tagged with Coumarin C6 (TCI) at a concentration of 2.5 µg/mL for 1 mL of the nanoparticle and were kept overnight in the dark. MCF7 cells were seeded over coverslip in 6 well plates at a density of 1 × 10^6^ cells/dish. The cells were then subjected to the C6 tagged AgNPs for 1 hr, and then the AgNPs were replaced with fresh media and finally incubated for 1 hr, 3 hr, and 6 hr, respectively. Post the Nanoparticle treatment, Mitotracker Red was added to the cells to stain the mitochondria of the cells and incubated for 20 min. The coverslips were washed with PBS thereafter were fixed with 4% Paraformaldehyde at room temperature, and mounted with DAPI, the anti-fading agent. The co-localization of AgNPs with Mitochondria was confirmed by visualizing the cells through ZEISS Axio Scope A1 Microscope, and the images were analyzed through ZEN 3.2 (blue edition) and Image J 1.53a software.

### 2.7 Measurement of ER tracker staining

To check the morphology of the Endoplasmic Reticulum of the cells, the MCF7 were seeded on the coverslips and were treated with AgNPs or drug plus AgNPs and incubated for 6 hr. After incubation, the cells were washed with PBS, and ER Tracker Green (Invitrogen) was added to the cells and incubated for 30–45 min at 37ºC and 5% CO2. The cells were then washed with PBS, and the slides were mounted and viewed under the microscope, as mentioned earlier.

### 2.8 Measurement of mitochondrial ROS production

The amount of Superoxide formed by mitochondria of certain cells can be determined by MitoSOX Red (Invitrogen). To check this Superoxide produced by the mitochondria of the cells, the MCF7 cells were seeded in a 6-well plate over a coverslip, and upon gaining their morphology, the cells were subjected to treatment for 1 hr, 3 hr, and 6 hr, respectively. After the drug incubation, 1 mL of 5 µM MitoSOX was added to each well and incubated for 10 min at room temperature. The cells were then washed with warm buffer thrice and fixed using Paraformaldehyde, and finally, the coverslips were mounted with the anti-fading agent, DAPI, and the cells were visualized under a fluorescent microscope (Zeiss, Axio Scope A1, or Axio Observer. Z1/7). ZEN 3.2 (blue edition) software was used to analyse the images.

### 2.9 JC-1 assay

To check the mitochondrial membrane potential upon the treatment of the AgNPs, JC-1 assay was performed where the cells were seeded in a 6-well plate with a seeding density of 60,000 cells/well, and they were allowed to adhere. After the cells gained their morphology, they were subjected to treatment for 6 hr. After treatment, the cells were stained with 75 nm JC-1 dye (working: 1 μl JC-1 from stock in 12 mL of complete DMEM) and incubated for 15–30 min at 37 ºC and 5% CO_2_ in the dark. The cells were harvested and resuspended in 1X PBS and immediately taken for flow cytometry analysis (Cytoflex, Beckmann Coulter), and the obtained data were analyzed using CytExpert Software.

### 2.10 Co-localization of ER and mitochondria

The MCF7 cells were seeded in a 6-well plate on coverslips and were allowed to adhere and grow for 24 hr. The cells were then treated with AgNP or a combination of AgNP with tunicamycin for 6 hr. Thereafter, the cells were incubated with Mito-tracker DeepRed and ER tracker Green respectively, one after the other, and the Mitochondria- ER co-localization was visualized under the fluorescent microscope (Zeiss, Axio Scope A1).

### 2.11 Cell viability assay

In vitro cell viability assay was performed using 3-[4,5-dimethyl-thiazol-2-yl]− 2,5-diphenyl tetrazolium bromide (MTT) dye. The MCF-7 cells were seeded at a density of 8 × 10^4^ cells per well in a 96-well plate, and the cells were incubated for 24 hr at 37ºC and 5% CO_2_ level. The cells were then exposed to AgNPs and drugs for 6 hr, and 24 hr, and the percentage of cell viability was observed. At each time interval, the medium was aspirated, and MTT dye was added to each well and incubated for 4 hr at 37ºC. The live cells form formazan crystals which were dissolved by adding DMSO (Dimethyl Sulfoxide), and the optical density was measured at 570 nm with the differential filter of 630 nm using a Spectrophotometer (Multiskan GO). The percentage cell viability was calculated using the formula- Viability (%) = (Mean value of drug-treated cells / mean absorbance of control cells) x 100.

### 2.12 Calcium uptake assay using Fura-2-AM

The cells were seeded in a 96-well plate at a density of 5000cells per well. Fura-2 was diluted in an appropriate buffer to make the final concentration between 0.1 and 5 µM. After the NP treatment, the cells were then incubated with Fura-2 for 30 min for esterification, after which the ester solution was removed, and the cells were washed and incubated with fresh buffer solution for de-esterification. The cells were incubated for another 30 min for the de-esterification process, and then the fluorescence activity was measured at the excitation maximum of 335 nm/360 nm and the emission maximum at 510 nm by using a microplate reader (Fluoroskan Ascent) [16].

### 2.13 Annexin V/PI staining

The MCF7 cells were seeded at a density of 5 × 10^5^ cells and were subjected to various treatments. After incubation, cells were harvested, washed with PBS, and resuspended in 500 μl of 1X binding buffer. After that, 4 μl of Annexin V-FITC and 10 μl of PI were added to the cells, respectively. Cells were then incubated for 15–20 min in the dark, and the samples were acquired using a flow cytometer (Cytoflex, Beckmann Coulter) and analysed using CytExpert software. Both early and late apoptotic cells were estimated by counting the percentage of cells in the lower and upper right (LR and UR) quadrant representative of only Annexin V and both Annexin V-PI positive cells, and the percentage of the cells was plotted using bar diagrams.

### 2.14 Statistical analysis

All experimental values were represented as mean ± SD. Unpaired t-test, one and two-way ANOVA were used for determining the statistical significance of data using Graph Pad Prism 8. The Bonferroni method was used for multiple comparisons. Data were considered statistically significant if p-value < 0.05. * or $ or # or @ or & indicates the statistically significance.

## 3 Results

### 3.1 AgNP localizes in the mitochondria and affects mitochondrial health

AgNPs were synthesized as per the procedure described earlier (Fig. 1a). The breast cancer cells- MCF7 were exposed to the AgNPs, and their internalization in a specific intracellular compartment like mitochondria was analyzed by confocal microscopy after stipulated time periods. To understand the selective localization of AgNPs in the mitochondria, live cells were treated with Mitotracker-Red, and the AgNPs were stained with Coumarin6 (C6)-Green, thereafter, the images obtained were merged and analysed for co-localization. We observed distinct yellow puncta predominantly at the peri-nuclear region, reflecting co-localization of AgNPs with mitochondria after 1-hr of exposure (Fig. 1b). Importantly, prolonged exposure to AgNPs resulted in internalization of the C6-tagged AgNPs in the DAPI blue-stained nucleus as well; however, no visible deformation of the nucleus was observed at the time point studied (Fig. 1b). Since mitochondria are the intra-cellular metabolic hub of the cell and play a key role in regulating redox homeostasis, we were therefore interested to understand whether the selective localization of the AgNPs has any impact on disruption of mitochondrial redox balance. A specific mitochondrial ROS staining dye-MitoSOX-red was used for this study [17]. Interestingly, the percentage shift of the red-fluorescence detecting peak in AgNP-treated cells, as analysed by flow cytometry, indicated an enhanced production of mitochondrial ROS. The level of ROS increased in a time-dependent manner with respect to untreated control (Fig. 1c). To further validate the generation of ROS in the mitochondria we performed confocal microscopy which also clearly indicates enhanced ROS generation after incubation of MCF7 cells with AgNPs (Fig. 1d). To analyse the resultant effect of mitochondrial localization of AgNPs we evaluated the putative alteration of mitochondrial membrane potential subsequent by JC1 staining (Fig. 1e). This dye, when it accumulates in healthy, negatively charged mitochondria, forms reversible J aggregates shifting its emission from green fluorescence to red; however, in cells with unhealthy mitochondria there is reduced formation of J aggregates thus the dye retains its primary green fluorescence. Importantly, after AgNP exposure, we observed a decrease in the red/green fluorescence intensity ratio reflective of a collapse of mitochondrial membrane potential (Fig. 1e).

### 3.2 AgNP induces mitochondrial fission

**Fig. 2:**
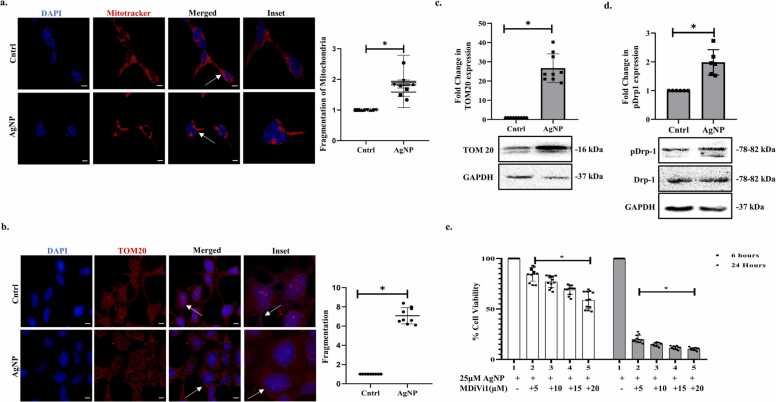
AgNP induces mitochondrial fission (a) Analysis of change in mitochondrial morphology post-AgNP (25 μM) exposure for 6 hr. Cells were stained with Mitotracker Red. Mitochondrial fragmentation was evaluated using Image-J software. Scale bar represents 10 µm. (b) Immunofluorescence study showing TOM20 protein expression post-AgNP (25 μM) treatment for 6 hr. Scale bar represents 10 µm. (c-d) Immunoblot representing expression of TOM20, pDRP1 & DRP1 post AgNP (25 μM) exposure for 6 hr. (e) Cell viability (MTT) assay post-treatment with different doses of MDiVi1 and/ or AgNP (25 μM). Data are expressed as mean±SD. * indicates the significant difference from control calculated using the student’s t-test (p < 0.05).

Mitochondria are the power hub of the cell, they regulate cellular metabolism, and calcium storage and show enormous plasticity in terms of their structure, localization, and function. While mitochondria can undergo active biogenesis, their morphology and number can be constantly regulated by contrasting processes, namely, fission and fusion. Therefore, cells actively maintain mitochondrial homeostasis as per their requirement, and such plasticity facilitates cellular adaptation to adverse conditions. In this context, AgNPs have been earlier reported to alter mitochondrial health, however, their effect on mitochondrial dynamicity is poorly elucidated. We observed that AgNP exposure resulted in an increase in the number of fragmented forms of mitochondria, as evident from analysis of mitochondrial morphology through Mitotracker Red staining in MCF7 cells (Fig. 2a); noteworthy in the untreated cells, the mitochondria were mostly elongated and connected compared to the NP-treated cells. Furthermore, we analysed the expression of the outer mitochondrial membrane (OMM) protein TOM20, which showed an even distribution in the cellular cytoplasm with prominent interconnections in the untreated cells; however, upon AgNP treatment, these interconnections were seemingly lost, and the localization was more clustered (Fig. 2b). Importantly, immunoblot analysis also showed an increase in the expression of TOM20 which might indicate that the fragmented mitochondrial state might be accompanied with augmented biogenesis and/or lack of clearance of damaged mitochondria by mitophagy (Fig. 2c). Thereafter to confirm triggering of mitochondrial fission by AgNPs, we evaluated the expression of the cytosolic GTPase- Dynamin Related Protein 1 (DRP1) which is known to be recruited to the OMM to facilitate GTP-dependent mitochondrial fission. AgNP exposure resulted in increased phosphorylation of DRP1, indicating its activation (Fig. 2d). Herein, to understand the role of mitochondrial fission induced by AgNPs, we inhibited DRP1 with the widely used inhibitor, MDiVi1 (mitochondrial division inhibitor). Inhibition of fission resulted in enhanced cytotoxicity of AgNPs at the time points studied, as analyzed by MTT assay, suggesting that induction of fission could be an adaptive cellular quality control strategy to minimize the cytotoxic effect of AgNPs (Fig. 2e).

### 3.3 AgNP induces ER stress

**Fig. 3:**
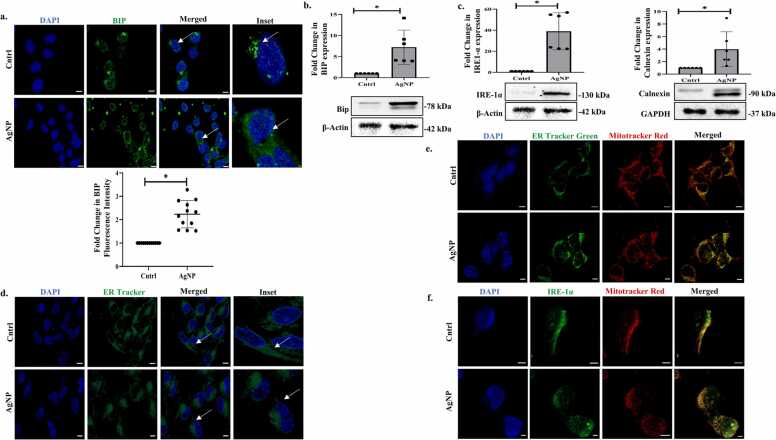
AgNP induces ER stress (a) Immunofluorescence images showing BIP post-AgNP (25 μM) treatment for 6 hr. Scale bar represents 10 µm. (b-c) Immunoblots representing the expression of BIP, IRE-1α & Calnexin post-AgNP (25 μM) exposure for 6 hr. (d) Images representing AgNP (25 μM) treated cells (6 hr) stained with ER tracker green. Scale bar represents 10 µm. (e) Images of co-localization of ER & mitochondria stained with ER tracker green & Mitotracker Red post-AgNP (25 μM) exposure for 6 hr. Scale bar represents 10 µm. (f) Immunofluorescence images showing co-localization of IRE-1α & Mitotracker Red post-AgNP (25 μM) exposure for 6 hr. Scale bar represents 10 µm. Data are expressed as mean±SD. * indicates the significant difference from control calculated using the student’s t-test (p < 0.05).

Cancer cells are in constant need of protein biogenesis as well as ATP production to maintain their proliferative needs. In this regard, ER is an important organelle that plays a vital role in protein synthesis and folding [18]. Interestingly, ER has also emerged as an organelle that communicates and regulates mitochondrial integrity and functionality as well. Mitochondria-associated ER membranes (MAMs) are structural domains that actively participate in maintaining various biological processes extending from calcium homeostasis, mitochondrial dynamics, ER stress to apoptosis [19]. However, this molecular crosstalk between mitochondria and ER is poorly understood. Considering our earlier observation that AgNPs can modulate mitochondrial function, we were hence interested to explore the effect of AgNPs on ER as well. Importantly, we observed an increase in the fluorescence intensity and protein expression of the ER resident, stress-sensor and chaperone- Bip or GRP78 (Fig. 3a and b). In addition, immunoblot analysis also showed an increase in expression of the calcium-binding chaperone protein- Calnexin, and also proteins involved in transduction of the unfolded protein response (UPR)- IRE1α triggered typically upon accumulation of misfolded proteins in the ER (Fig. 3c). Proteins like IRE1α, when bound to Bip, remains inactive, however, with the induction of ER stress, Bip frees itself from the conjugation leading to the activation of IRE1α that helps in reducing the burden of misfolded protein and overall protein load thus restoring cellular homeostasis [20]. Furthermore, existing studies show that with the induction of ER stress, the localization and the fine tubular structure of ER can be disrupted. In corroboration to above, staining of AgNP-treated cells with ER Tracker Green showed a re-distributed, more concentric perinuclear localization of ER in the AgNP-treated cells compared to untreated control (Fig. 3d). As discussed earlier, there exists physical-functional coupling between mitochondria and ER [21]; interestingly, staining of ER with ER-tracker-Green and mitochondria with Mito-tracker-DeepRed revealed extensive overlapping regions which considerably increased in AgNP treated cells, confirming their increased contacts under stressed conditions (Fig. 3e). This was further confirmed through immuno-staining of ER membrane-protein- IRE1 alpha and its spatial arrangement with respect to stained mitochondria (Fig. 3f). The above results clearly indicate that AgNP triggers adaptive response in both mitochondria and ER, and they possibly act in concert to restore homeostasis or dictate cellular fate.

### 3.4 Enhancement of AgNP-induced ER stress affects mitochondria and cell fate

**Fig. 4:**
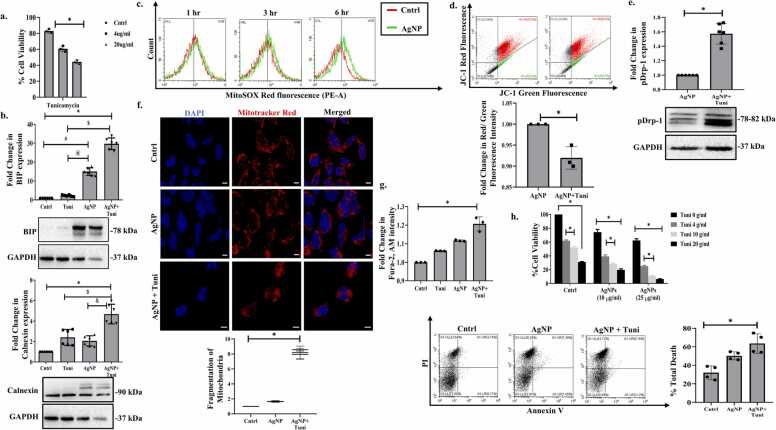
Enhancement of AgNP-induced ER stress affects mitochondria & cell fate (a) Analysis of cytotoxicity post-treatment (24 hr) of different doses of ER stress inducer Tunicamycin. (b) Immunoblot representing the expression of BIP & Calnexin post-AgNP (25 μM) and/ or tunicamycin (10 μg/mL) exposure for 6 hr. (c) Analysis of mitochondrial ROS using MitoSOX-red upon AgNP (25 μM) and/or tunicamycin (10 μg/mL) exposure as analyzed through flow cytometry. (d) Analysis of mitochondrial membrane potential by JC-1 post AgNP (25 μM) and/ or tunicamycin (10 μg/mL) treatment for 6 hr. (e) Immunoblot representing the expression of pDRP1 post AgNP (25 μM) &/or tunicamycin (10 μg/mL) exposure for 6 hr. (f) Analysis of mitochondrial fragmentation post AgNP (25 μM) and/or tunicamycin (10 μg/mL) treatment (6 hr) through Mitotracker Red staining. Mitochondrial fragmentation was evaluated using Image-J software. Scale bar represents 10 µm. (g) Analysis of intra-cellular Ca^+2^ levels post AgNP (25 μM) and/or tunicamycin (10 μg/mL) treatment (6 hr) using Fura-2, AM. (h) Analysis of cell death in AgNP (25 μM) and/or tunicamycin (10 μg/mL) treated cells as analysed through MTT assay & Annexin V-PI staining. * , #, &, @ indicates the significant difference from control & different groups, calculated using the student’s t-test and one-way Annova with Bon-ferroni post-test (p < 0.05).

As discussed earlier, mitochondria and ER are interdependent organelles and experience physical-functional coupling that can be of biological significance under cellular stress. Earlier reports suggest the transfer of Ca^2+^ between organelles or augmentation of mitochondrial metabolic activity/ATP generation through such avenues. Herein, we were interested to understand the crosstalk between induction of ER stress and mitochondrial dynamics upon AgNP exposure. We used tunicamycin, a pharmacological agent that stimulates the accumulation of misfolded proteins to enhance AgNP-induced ER stress and thereafter monitor mitochondrial dynamics and associated cell fate [22]. The dose of tunicamycin was standardized through MTT assay (Fig. 4a), and simultaneous treatment with AgNP caused a significant increase in expression of the ER stress proteins- Bip and calnexin (Fig. 4b). Importantly, the enhancement of ER stress with tunicamycin was accompanied with a time-dependent increase in the production of mitochondrial ROS (Fig. 4c) and an increase in mitochondrial membrane depolarization suggesting that an enhancement of ER stress directly affects mitochondrial health (Fig. 4d). Interestingly, an increase in ER stress with tunicamycin was also associated with an elevated protein level of phospho-DRP 1 (pDRP1) indicative of enhanced mitochondrial fission (Fig. 4e). Increased fragmentation of mitochondria in the presence of AgNP and tunicamycin was also evident from Mitotracker staining (Fig. 4 f) clearly indicating that enhancement of AgNP-induced ER stress triggers increased mitochondrial fission. Herein, ER is the primary site for intra-cellular Ca^2+^ storage and overloading of Ca^2+^ in proximity to mitochondria can often result in Ca^2+^ uptake by mitochondria and stimulation of mitochondria-driven cell death [23]. Importantly, we have earlier observed that AgNP treatment, and also AgNP plus tunicamycin (data not shown), results in the increased structural overlap between ER and mitochondria. Our results further depict that there is a considerable increase in intra-cellular Ca^2+^ levels, as analyzed through Fura-2 staining upon exposure of MCF7 cells to AgNP, or the nanoparticle plus tunicamycin indicating a probable role of Ca^2+^ in dictating cell fate (Fig. 4g). Importantly, both MTT assay and Annexin V/PI staining confirmed a stimulation of cell death in AgNP and tunicamycin-treated cells suggesting that induction of ER stress is plausibly associated with increased cytotoxicity (Fig. 4h). Overall, we show that enhancement of AgNP-induced ER stress can result in augmented mitochondrial fission, increase in cytosolic Ca^2+^ levels and subsequent induction of cell death. Further studies are required in this direction to delineate the precise mechanism involved.

## 4 Discussion

In recent years, there has been a revolution in cancer drug development with a major emphasis on targeted or personalized therapy. However, the cost of such therapeutic measures is often exorbitant and is, therefore, beyond the reach of many cancer patients. Therefore, chemotherapy still plays a major role in cancer treatment, either administered alone or in combination with other forms of therapy. In this regard, especially for cancers with no targeted therapy available [24], [25], standard chemotherapy is still the current gold standard of treatment [26], [27]. The success of conventional chemotherapy is majorly hindered by the acquisition of drug resistance resulting in increased complexity of the treatment regimen [28]. In this context, nanotechnology has emerged as a novel alternative approach to conventional therapies. Recently, various studies on nano-formulations have been reported. *Dorjsuren* et al. came up with a combination therapy of Cetuximab-coated thermo-sensitive liposome-loaded magnetic NPs against breast cancer [29]; also, metal NPs are reported to offer diverse opportunities owing to their suitable physicochemical and biological properties [30]. Herein, the use of iron-doped titanium oxide nanodots by *Bai* et al. [24], ZnPc/protein nanohorns by *Zhang* et al. [25], antibody-phthalocyanine-gold NPs by *Stuchinskaya* et al. against breast cancer [26], Fe_3_O_4_ core–TiO_2_ shell nanocomposites and nanoconjugates by *Liu* et al. for neuroblastoma radio-senitization [27] are few of the many promising ones that have emerged as an alternative mode of therapy. In this regard, there has been much progress in the use of gold, titanium and silver NPs as potential therapeutics against multiple cancer types. However, a better understanding of their intracellular mechanism of action remains elusive, which can further be explored to accelerate their probable use in the field of therapeutics. In this study, we show how AgNPs affect organelle dynamics and how the crosstalk between the organelles determines breast cancer cell fate. Herein, mitochondria are essential sub-cellular organelles which are considered for drug targeting due to their important roles in cell proliferation and death as well. They perform a variety of important cellular functions, including regulation of programmed cell death [6], [7], [8], [9], [10] and cellular ATP production. Moreover, oxidative metabolism, which occurs in cancer cells [13], results in the production of a high amount of reactive oxygen species (ROS), and these elevated ROS levels activate signaling pathways that promote cell proliferation and tumorigenesis. In addition, mitochondria are the central regulators of cellular metabolism and the mediators of apoptosis [23]. Since the majority of the cancer cells have suppressed apoptosis, NPs designed to trigger mitochondrial de-homeostasis are likely to be a promising strategy for sensitizing cancer cells. We show that AgNPs preferentially localize in the mitochondria, enhancing the production of mitochondrial ROS coupled to an increase in the mitochondrial membrane depolarization resulting in stimulation of mitochondrial fission in breast cancer cells. Our studies corroborate earlier findings where mitochondria-targeting drugs, along with conventional cancer therapeutics like paclitaxel/cisplatin, efficiently induced cytotoxicity through mitochondrial damage and release of cytochrome C [31]. Similarly, *Xu* et al. have also shown that the green biosynthesized AgNPs from *Ginkgo biloba* extracts can induce apoptosis in the cancer cells by generating ROS and by causing mitochondrial dysfunction [32]. There are many other studies as well that highlight the potential of metal NPs disrupting mitochondrial homeostasis, which can be capitalized on for their future use in therapeutics.

Another important organelle that is intricately linked to cancer cell function is ER. It is one of the vital organelles primarily involved in protein synthesis, folding, transport, and lipid biosynthesis. However, in cancer cells, its functions are often dysregulated, leading to ER stress. There are existing reports that establish the crosstalk between ER and mitochondria. According to *Sassano* et al., the first-ever inter-organelle contact site was discovered was the entanglement (mitochondria-associated membranes) between mitochondria and ER [30], and they proposed that inter-organelle Ca^+2^ ion flow maintains their functionality [33]. Miscommunication between these organelles can lead to cellular bio-chemical dis-equilibrium shifting balance towards disorders [31]. Herein, a few studies show that induction of ER stress by NPs can impair cancer cell progression [31], [34], [35], [36]. For example, AgNPs synthesized by *Simard* et al. mediated ER stress and subsequent apoptosis in breast cancer cells [37]; similarly, other research groups have also targeted both ER and mitochondrial activity by nano-formulations [38], [39]; however, the crosstalk between these two critical organelles in response to AgNP exposure is poorly understood. Importantly, in this study, we show that AgNPs, apart from altering mitochondrial health, can also significantly disrupt the tubular nature of ER morphology and reissuances stress markers like GRP78, IRE 1 alpha, and Calnexin [22]. Interestingly, an earlier study by *Pandey* et al. showed that ER and mitochondria-targeted NPs could potentially inhibit the function of anti-apoptotic proteins like Bcl2 facilitating cell death, indicating the importance and putative synergy between these organelles, which can be utilized in the future for cancer therapy [40]. On a similar note, to understand the ER-Mito crosstalk, we increased ER stress with a well-known ER stress inducer- Tunicamycin, alongside AgNP treatment, and it resulted in an enhanced de-regulation of mitochondrial homeostasis marked by increased mitochondrial ROS and fragmentation, leading to cytotoxicity. This further confirmed the interlinked function of mitochondria and ER in tumor cells. Our study thus shows how AgNPs disrupt the health of key organelles critical to cancer cell function, which can be further developed for future cancer therapy.

## Funding

SD is a recipient of an SRF fellowship from 10.13039/501100001411ICMR, India project (Grant: 5/12/15/2019/NCD-III). AS is a recipient of an Institute fellowship from 10.13039/501100006464BITS Pilani. This work was supported in part by ICMR, India grants to RC (2020-1404/ADHOC-BMS) and SC (Grant number: 5/12/15/2019/NCD-III).

## Declaration of Competing Interest

The authors declare that they have no known competing financial interests or personal relationships that could have appeared to influence the work reported in this paper.

## Data Availability

Data will be made available on request.
